# Damping Estimation from Free Decay Responses of Cables with MR Dampers

**DOI:** 10.1155/2015/861954

**Published:** 2015-06-17

**Authors:** Felix Weber, Hans Distl

**Affiliations:** ^1^Structural Engineering Research Laboratory, Swiss Federal Laboratories for Materials Science and Technology (Empa), Überlandstrasse 129, 8600 Dübendorf, Switzerland; ^2^Maurer Söhne GmbH & Co. KG, Frankfurter Ring 193, 80807 München, Germany

## Abstract

This paper discusses the damping measurements on cables with real-time controlled MR dampers that were performed on a laboratory scale single strand cable and on cables of the Sutong Bridge, China. The control approach aims at producing amplitude and frequency independent cable damping which is confirmed by the tests. The experimentally obtained cable damping in comparison to the theoretical value due to optimal linear viscous damping reveals that support conditions of the cable anchors, force tracking errors in the actual MR damper force, energy spillover to higher modes, and excitation and sensor cables hanging on the stay cable must be taken into consideration for the interpretation of the identified cable damping values.

## 1. Introduction

Observations on long free span stay cable bridges show that stay cables are susceptible to large amplitude vibrations because of their low inherent damping and length of several hundred meters. Well-known examples are the Dongting Lake Bridge in China [1], the Franjo Tudjman Bridge in Croatia [2], and the Alamillo Bridge in Spain [3]. The excitation mechanisms are based on combined rain-wind and dry wind effects [4–7] or parametric excitation [8]. In order to make cables safe against these disturbing mechanisms, the logarithmic decrement of cables should be at least 3% to 4% according to the fib bulletin 30 [9]. This can be achieved by the very effective but also costly solution of hydraulic actuators that are installed in cable axis [10] or by transverse dampers which represent the commonly adopted solution since transverse cable dampers represent more economical solution than the active tendons and can also be installed as retrofit measure.

The design of transverse cable dampers is based on the design of the linear viscous damper. Its design parameter is the viscous damper coefficient which can be optimally tuned for maximum damping of one mode of vibration [11–13]. The usual requirement on real stay cable bridges is to mitigate the first three or four cable modes by the transverse damper. Then, the viscous damper coefficient is designed to a frequency which is between the lowest and highest eigenfrequencies of the targeted modes. In order to ensure sufficient damping according to [9] in each targeted mode with this design, the damper position must be increased compared to the situation of one targeted mode [14]. However, the increased damper position may undesirably affect the aesthetics of the bridge and tall damper supports may be costly. Another approach to provide sufficient damping in several targeted modes by transverse dampers is the adoption of passive friction dampers [15]. The drawback of this approach is that passive friction dampers evoke amplitude dependent cable damping whereby the damping at small and large cable amplitudes may become insufficient [16, 17].

The need to generate cable damping by transverse dampers which is independent of the actually vibrating mode, that is, the actual frequency of vibration, and is independent of the actual cable displacement amplitude triggered the development of controlled stay cable damping systems of which most are based on semiactively controlled magnetorheological (MR) dampers due to their suitable force range and fail-safe behaviour. The authors of [18–21] showed that MR dampers operated in the passive mode, that is, at constant current, yield amplitude dependent cable damping due to the predominant friction behaviour of the actual MR damper force at constant current. As shown in [17, 22, 23], this can be avoided by controlling the friction force level in proportion to the collocated displacement amplitude. Another strategy is to emulate viscous damping with MR dampers where the viscous damper coefficient is adjusted to the actual frequency of vibration [24, 25]. Both approaches evoke frequency and amplitude independent cable damping and the resulting cable damping is equal to the value due to optimal viscous damping of a taut string [12]. In order to make full use of the controllability of MR dampers, the damping force must be shaped by a superimposed negative stiffness force which is obtained from the clipped optimal control approach [26–31], from amplitude proportional friction damping with negative stiffness [32] or clipped viscous damping with negative stiffness [33, 34]. As shown in [32], the approaches [26–34] are able to produce twice as much damping in a taut string as optimal viscous damping due to the negative stiffness emulation by the semiactive MR damper force [35, 36].

The verification of the damping efficiency of the aforementioned control strategies on real stay cables with transverse MR dampers is commonly realized by free decay tests from which the cable damping is assessed by the logarithmic decrement method [20, 37, 38]. Since these tests are conducted on real stay cables, it is reasonable to compare the obtained cable damping values not only to the theoretically achievable value due to optimal viscous damping of a taut string [12], that is, a cable without flexural rigidity and simply supported ends, but also to the theoretically achievable value due to optimal viscous damping of a cable with typical flexural rigidity and fixed supported ends [39–42] which is the aim of this paper. Free decay tests on three different cables with different anchors and cycle energy controlled (CEC) MR damper are described. The comparison of the obtained cable damping values to the above mentioned theoretic benchmark values shows that support conditions, force tracking errors in the actual MR damper force, energy spillover to higher modes, and the presence of excitation and sensor cables must be taken into consideration for the interpretation of the results.

The layout of the paper is as follows.  Section 2 introduces transverse damping of a taut string by viscous and energy equivalent friction dampers based on which the CEC approach for MR dampers is derived.  Section 3 describes the experimental validation of the CEC approach on a single strand cable and discusses the obtained cable damping results. The same is done in Section 4 for a CEC controlled stay cable of the Sutong Bridge with provisional anchors and a CEC controlled stay cable on the Sutong Bridge with common anchors. The paper is closed by a short summary with concluding remarks.

## 2. Control of Friction Type Dampers

### 2.1. Passive Viscous Damper

**Figure 1:**
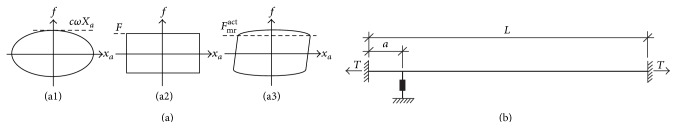
Force displacement trajectories (a) of linear viscous damper (a1), passive friction damper (a2), and MR damper at constant current (a3); taut string with transverse damper (b).

The prototype damper is the passive viscous damper whose force *f* is proportional to its velocity ${\dot{x}}_{a}$:

(1) $$\begin{array}{c} f=c{\dot{x}}_{a}, \end{array}$$

where *c* denotes the damper viscous coefficient (Figure 1(a1)). If this damper type is used as transverse cable damper at the distance *a* from the left support of a taut string with length *L*, tension force *T*, and radial frequency *ω* of the vibrating mode (Figure 1(b)), *c* must be selected as follows [11–13]:

(2) $$\begin{array}{c} c=\frac{T}{a\omega } \end{array}$$

to maximize the theoretically attainable cable damping which is given here by the logarithmic decrement

(3) $$\begin{array}{c} \delta^{\text{theo}}≅\pi \frac{a}{L}. \end{array}$$

Due to *c* ~ *ω* ^−1^ in (2), viscous dampers can maximize the cable damping in one mode only. If several modes are to be mitigated, as it is typically required for real stay cable bridges [9], *c* is designed to a frequency between the lowest and highest eigenfrequencies of the targeted modes [14]. In order to secure that the damping in each targeted mode of the cable with transverse damper is equal to or greater than the recommended value of 3% to 4% to make the cables safe against rain-wind induced vibrations [9], the relative damper position *a*/*L* must be increased compared to the situation when only one mode is targeted. However, increased damper position undesirably affects the aesthetics of the bridge and is costly due to tall damper supports.

### 2.2. Passive Friction Damper

The other prototype damper is the passive friction damper. The simplest friction model is the Coulomb friction approach

(4) $$\begin{array}{c} f=F\mathrm{sgn}\left({\dot{x}}_{a}\right), \end{array}$$

where *F* denotes the friction force amplitude (Figure 1(a2)). Balancing the cycle energies of passive viscous and friction dampers yields

(5) $$\begin{array}{c} \pi c\omega X_{a}^{2}=4FX_{a}, \end{array}$$

where *X*_*a*_ is the damper displacement amplitude. The friction force amplitude *F* for maximum cable damping is obtained by the substitution of (2) into (5):

(6) $$\begin{array}{c} F=\frac{\pi }{4}\frac{T}{a}X_{a}. \end{array}$$

Expression (6) shows that passive friction dampers maximize the cable damping for one damper amplitude and consequently for one cable amplitude only [16, 17], but the tuning of *F* does not depend on *ω* and thereby does not depend on the mode of vibration. In order to overcome the drawback of frequency dependent cable damping in case of passive viscous dampers and amplitude dependent cable damping in case of passive friction dampers, two possible approaches exist to generate frequency and amplitude independent cable damping: dampers with controllable *c* [24, 25] and dampers with controllable *F* of which the latter approach is described next.

### 2.3. Controlled Friction Type Damper

According to (6), the desired force *f* ^*des*⁡^ of a controlled friction damper that generates frequency and amplitude independent cable damping becomes

(7) $$\begin{array}{c} f^{\mathrm{des}}=\mathrm{sgn}\left({\dot{x}}_{a}\right)\left(\frac{\pi }{4}\frac{T}{a}\right)X_{a} \end{array}$$

which is fully dissipative because of $f^{\mathrm{des}}\sim \mathrm{sgn}\left({\dot{x}}_{a}\right)$. Thus, a semiactive device such as a controllable damper is able to emulate (7). Due to the big required damping forces for stay cable mitigation by transverse dampers at damper positions in the range of 1% to 4%, semiactive MR dampers represent the appropriate choice to track (7). However, as the analysis of a controlled friction damper on a taut string in [17] shows, the controlled friction force (7) evokes almost rectangular shaped cable displacement at damper position which leads to large curvatures and consequently high bending stresses in the cable at damper position which eventually may lead to premature material fatigue. Hence, (7) does not represent the optimal solution for stay cable damping.

**Figure 2:**
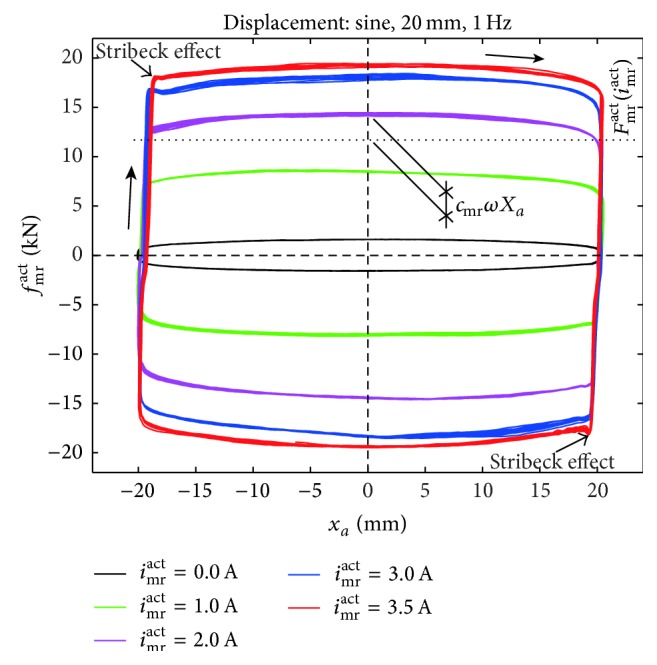
Force displacement trajectories of MR damper force at constant current.

In order to derive a control law that avoids premature material fatigue on the cable at damper position, it is worth looking at the force displacement trajectories of MR dampers at constant current (Figure 2). It is seen from Figure 2 that the actual MR damper force *f*_mr_^act^ can be described as the superposition of the strongly current dependent friction force amplitude *F*_mr_^act^ and the weakly current dependent viscous force component; thus,

(8) $$\begin{array}{c} f_{\text{mr}}^{\text{act}}=F_{\text{mr}}^{\text{act}}\mathrm{sgn}\left({\dot{x}}_{a}\right)+c_{\text{mr}}{\dot{x}}_{a}, \end{array}$$

where *c*_mr_ denotes the MR damper viscous coefficient and the preyield stiffness of *f*_mr_^act^ is neglected (Bingham model). Considering that *F*_mr_^act^ is a strong function of the actual MR damper current *i*_mr_^act^, the proposed control approach here aims at

- controlling *F*_mr_^act^ in proportion to *X*_*a*_,
- defining the desired value of *F*_mr_^act^ such that the cycle energy of *f*_mr_^act^ is equal to the cycle energy of optimal viscous damping ((2), Figure 1(a3)) to ensure maximum cable damping (3).

Thus, the desired friction force amplitude *F*_mr_^*des*⁡^ to be tracked in real-time by *F*_mr_^act^ of the MR damper is

(9) $$\begin{array}{c} F_{\text{mr}}^{\mathrm{des}}=\frac{\pi }{4}X_{a}\left\{\frac{T}{a}-c_{\text{mr}}\omega \right\} \end{array}$$

which ends up in* constant actual current i*_mr_^act^ as long as *X*_*a*_ does not change and varies only a little from cycle to cycle during cable resonant vibrations since then cable amplitudes and consequently *X*_*a*_ change slowly over time. The resulting force displacement trajectories therefore do include the small viscous force component $c_{\text{mr}}{\dot{x}}_{a}$ as depicted in Figure 2 whereby the cable displacement at damper position is not rectangular shaped as for pure friction damping. Since *F*_mr_^act^ is controlled in proportion to *X*_*a*_, the resulting cable damping is independent of the actual cable amplitude. Due to the existent but small viscous component $c_{\text{mr}}{\dot{x}}_{a}$, the resulting cable damping is not fully but almost independent of the actual cable frequency. The control law (9) is called cycle energy control (CEC) approach because it leads to the same energy dissipation as optimal viscous damping (5); detailed information on CEC is available in [23].

## 3. Cable Damping Tests on Laboratory Scale Cable

### 3.1. Cable Damper Set-Up at Empa

**Figure 3:**
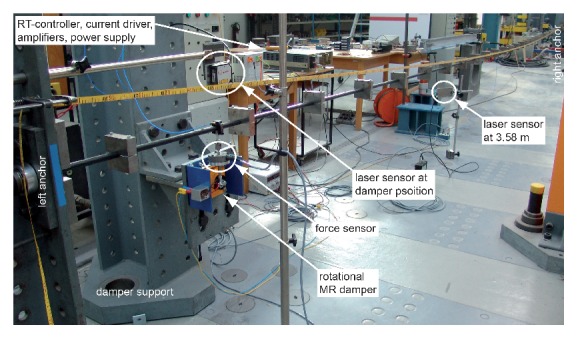
Cable damper test set-up at Empa.

The CEC approach was experimentally tested with a rotational MR damper on a steel wire strand of length *L* = 16.54 m and tensioned at 22 kN. Its mass per unit length *m* including the additional masses visible in Figure 3 was 5.85 kg/m; the resulting fundamental frequency *f*_1_ was 2.0 Hz. The damper was positioned at *a* = 0.66 m which corresponds to 4% of the cable length. The MR damper under consideration showed a pronounced friction force behaviour at constant current with a residual force of approximately 13 N and a maximum force of approximately 167 N at 3.5 A while the viscous force component was negligibly small; that is, *c*_mr_ ≈ 0 Ns/m. More information on this rotational MR damper and its working behaviour is available in [43]. The actual cable displacement at damper position *x*_*a*_ was measured by a laser triangulation sensor which was used as the only input to the real-time controller (dSPACE) to compute *X*_*a*_ based on the latest amplitude and the desired control force (9). The corresponding desired MR damper current *i*_mr_^*des*⁡^ was estimated from a mapping approach [23]. The amplifier of type KEPCO was adopted as current driver to ensure *i*_mr_^act^ ≈ *i*_mr_^*des*⁡^. The actual MR damper force was measured by a 500 N load cell between cable and damper rod to double-check the force tracking accuracy.

### 3.2. Cable Damping Assessment

The cable damping is assessed from free decay curves logarithmic decrement method [37, 38]:

(10) $$\begin{array}{c} \delta^{\text{mea}}\left(t_{i}\right)=\ln\left(\frac{X_{c}\left(t_{i}\right)}{X_{c}\left(t_{i}+T_{d}\right)}\right), \end{array}$$

where *X*_*c*_(*t*_*i*_) denotes the amplitude at time instant *t*_*i*_ of the cable displacement *x*_*c*_ that was measured by a laser triangulation sensor at 3.58 m from the left anchor and *T*_*d*_ is the damped time period. The free decay data is evaluated both from peak to peak, which is denoted as the point-to-point logarithmic decrement and by the exponential fit of the peaks during the decay to derive the best average estimate of the logarithmic decrement.

### 3.3. Test Results

**Figure 4:**
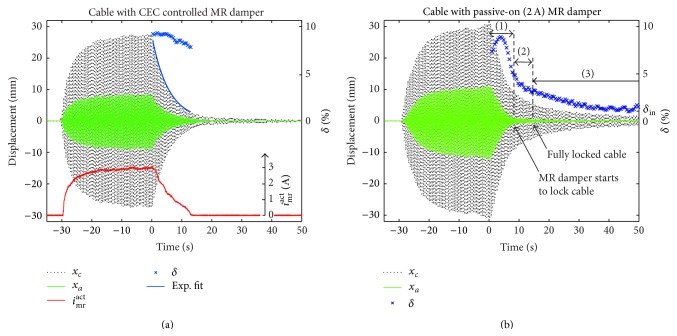
Free decay curve with CEC controlled (a) and passive-on (b) MR damper.

The cable was* excited directly by hand* at its fundamental eigenfrequency* at midspan*. The logarithmic decrement of mode 1 is determined when the MR damper force is controlled by the CEC approach (9) and when the MR damper is operated in the passive mode, that is, at constant *i*_mr_^act^, as benchmark. As observed from Figure 4(a), the point-to-point logarithmic decrement hardly varies during the free decay whereby the decay envelope is exponentially shaped when the CEC approach is adopted. Hence, the cable damping is independent of amplitude as targeted by CEC. The measured mean logarithmic decrement ${\overset{-}{\delta }}^{\text{mea}}$ of 8.73% divided by the theoretically attainable value *δ* ^theo^ (3) yields the damping efficiency *η* as follows:

(11) $$\begin{array}{c} \eta =\frac{{\overset{-}{\delta }}^{\text{mea}}}{\delta^{\text{theo}}} \end{array}$$

whose average value of all (four) CEC tests is approximately 70%. The efficiency loss of approximately 30% relative to optimal viscous damping (2) is explained by the cable flexural rigidity and fixed supported ends of the strand under consideration whereby approximately only 80% of *δ* ^theo^ can be expected [39]. This loss of approximately 20% is also confirmed by the difference of approximately 19% at 4% of the cable length [40] between the mode shapes of a cable without flexural rigidity and simply supported ends and a cable with flexural rigidity and fixed supported ends. The remaining 10% of losses are interpreted as the sum of losses due to force tracking errors and due to the small bearing play (which is mandatory!) of both hinged joints of the damper that reduce the effective damper motion.

When the MR damper is operated in the passive mode (Figure 4(b)), the point-to-point logarithmic decrement strongly depends on amplitude due to the uncontrolled, that is, constant, friction force amplitude *F*_mr_^act^ as long as the damper works (section 1). Within section 2, the constant friction force amplitude *F*_mr_^act^ starts to lock the cable at damper position that is confirmed by *x*_*a*_. At the beginning of section 3, the cable can be considered to be fully locked by the uncontrolled *F*_mr_^act^ whereby the cable vibrates as the case without external damper and *δ* ^mea^(*t*_*i*_) converges towards the inherent logarithmic decrement *δ* ^in^ ≈ 1%.

**Figure 5:**
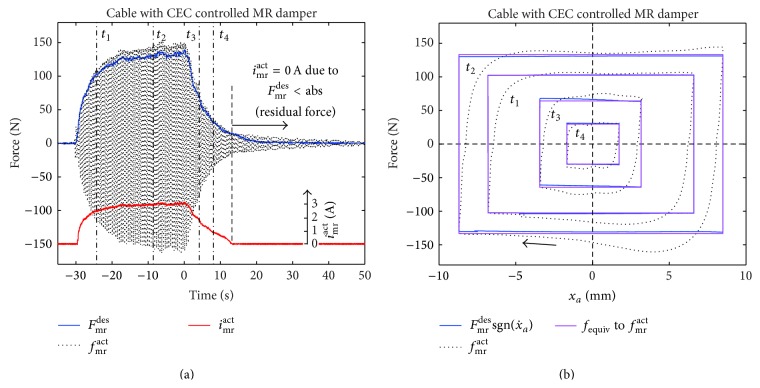
Desired friction force, actual MR damper force, and energy equivalent friction force of actual MR damper force.

The real-time force tracking depicted in Figure 5(a) shows that *F*_mr_^act^ is smaller than max⁡(*f*_mr_^act^), due to the peak force which occurs at the end of each half damper cycle; see Figure 5(b). The force tracking accuracy is assessed by the comparison of *F*_mr_^*des*⁡^ multiplied by $\mathrm{sgn}({\dot{x}}_{a})$ (measured ${\dot{x}}_{a}$) to get the blue force displacement trajectories with the friction force *f*_equiv_ which is the energy equivalent of the actual (measured) MR damper force *f*_mr_^act^ and computed as follows:

(12) $$\begin{array}{c} f_{\text{equiv}}=\mathrm{sgn}\left({\dot{x}}_{a}\right)\left\{\frac{1}{4X_{a}}\overset{T_{d}}{\underset{0}{\int }}f_{\text{mr}}^{\text{act}}{\dot{x}}_{a}dt\right\}. \end{array}$$

The numerical comparison of *f*_equiv_ with $F_{\text{mr}}^{\mathrm{des}}\mathrm{sgn}({\dot{x}}_{a})$ within the four selected time windows yields the average force tracking error of 5.72%. This fairly small force tracking error is the direct result of the CEC approach which ends up in *i*_mr_^act^ = constant during each half cycle in contrast to, for example, the emulation of viscous damping that requires fast modulation of *i*_mr_^act^ during each half cycle and consequently is more susceptible to force tracking errors [24, 25, 43].

**Figure 6:**
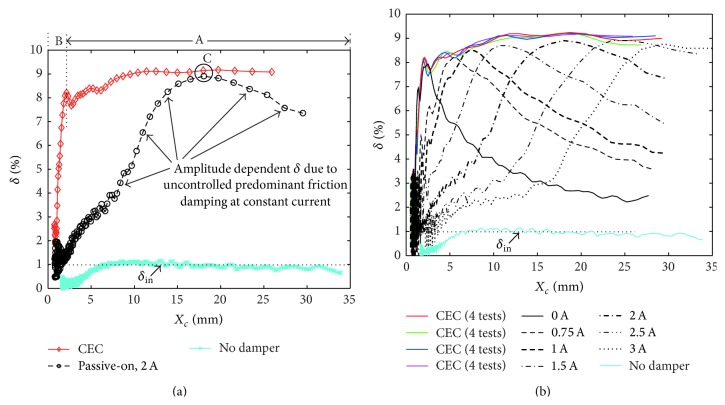
Logarithmic decrement versus cable displacement amplitude.

The values of *δ* ^mea^(*t*_*i*_) are plotted versus the values of *X*_*c*_(*t*_*i*_) in Figure 6(a) for the CEC approach and the passive mode. This representation points out the following.

- The CEC approach approximately yields amplitude independent cable damping as long as *F*_mr_^*des*⁡^ is not constrained by the residual force at 0 A (section A in Figure 6(a)).
- When *F*_mr_^*des*⁡^ is constrained by the residual force, *δ* ^mea^(*t*_*i*_) drops towards *δ* ^in^ (section B). The cable can be considered to be fully locked by the residual force when *δ* ^mea^(*t*_*i*_) ≈ 1% where *X*_*c*_(*t*_*i*_) ≤ 1 mm and *X*_*a*_(*t*_*i*_) ≤ 0.17 mm, that is, when cable amplitudes are negligibly small. The scattering of *δ* ^mea^ visible in Figure 6 at small cable amplitudes *X*_*c*_ < 2.5 mm and therefore *X*_*a*_ < 0.3 mm results from higher modes in the cable which are triggered when the MR damper starts to lock the cable whereby the peaks become modulated and consequently *δ* ^mea^ is scattered. This could be avoided by stronger bandpass filtering of the measured cable displacement *x*_*c*_ which is not done here in order to show the true cable response.
- The passive mode of the MR damper evokes strongly amplitude dependent *δ* ^mea^(*t*_*i*_) due to the uncontrolled, that is, constant, friction force amplitude *F*_mr_^act^. At point C *δ* ^mea^(*t*_*i*_) due to 2 A and *δ* ^mea^(*t*_*i*_) due to CEC are approximately equal because *F*_mr_^act^ resulting from 2 A is correctly tuned to the actual damper amplitude which occurs when *X*_*c*_ ≈ 18 mm.

Figure 6(b) plots the curves of all passive mode tests and the results of all (four) CEC tests. It is seen that the CEC approach tracks the maxima of the curves due to passive mode which proves that the gain (*πT*)/(4*a*) in (9) is correct.

## 4. Cable Damping Tests with Cables of Sutong Bridge

### 4.1. Cable Damping Tests at Fasten Nippon Steel, China

The CEC approach was developed at Empa in 2004/2005 and tested by hybrid simulations at Empa in 2006 with a cylindrical MR damper as later installed on the Sutong Bridge. In 2007, this adaptive stay cable damping system was tested by the authors on a 228 m long stay cable at Fasten Nippon Steel, China, which is described subsequently.

#### 4.1.1. Test Set-Up

**Figure 7:**
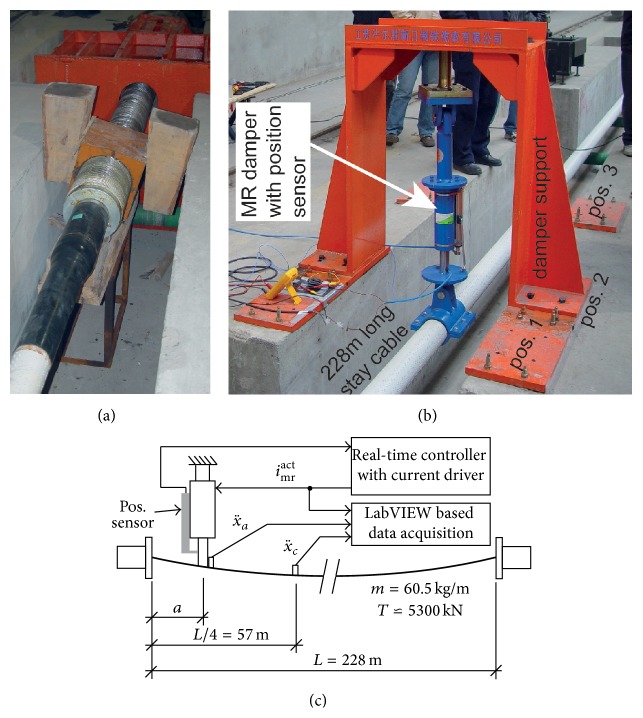
Cable damping tests at Fasten Nippon Steel, China: provisional anchor of test cable (a); tested damper positions (b); sketch of test set-up (c).

The stay cable was of the same type as that installed on the Sutong Bridge. The cable was anchored at its both ends by provisional anchors, one of which is depicted in Figure 7(a). The force characteristics of the cylindrical MR damper visible in Figure 7(b) are those shown in Figure 2 where the current dependent friction force amplitude *F*_mr_^act^ is highlighted by the horizontal dashed line and *c*_mr_ of approximately 24.7 kNs/m must not be neglected. The relevant test cable properties are given in Figure 7(c) which resulted in *f*_1_ ≈ 0.65 Hz. The cable was excited* directly by hand at the nodal point of the targeted mode.* The cable responses were measured at quarter span and damper position with accelerometers and acquired with an 8-channel IO-board of NI using the software LabVIEW. The real-time controller, current driver, and position sensor used for these tests were of the same type as that later installed in the Sutong Bridge. The cable damping was measured for

- the three damper positions 5.95 m (*a*/*L* = 2.61%), 6.56 m (*a*/*L* = 2.88%), and 7.81 m (*a*/*L* = 3.43%),
- modes 1, 2, and 3,
- the CEC controlled MR damper,
- the MR damper in passive-off (0 A) mode supposing a power breakdown of the mains and, at the same time, a power breakdown of the accumulators that can provide the required power for 48 h.

#### 4.1.2. Test Results

**Figure 8:**
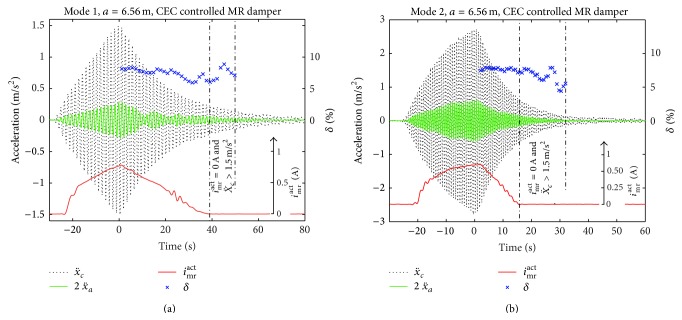
Free decay tests with CEC controlled MR damper at damper position *a* = 6.56 m for mode 1 (a) and mode 2 (b).

**Figure 9:**
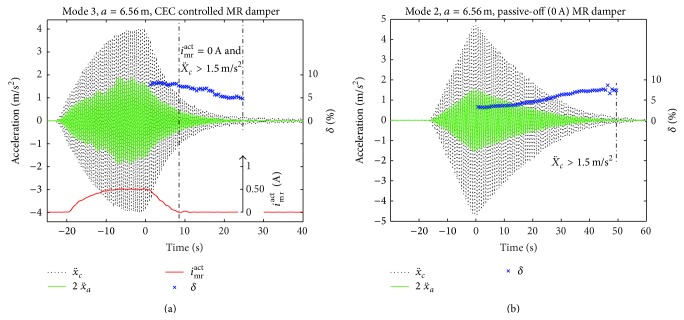
Free decay tests with CEC controlled MR damper at damper position *a* = 6.56 m for mode 3 (a) and with passive-off (0 A) MR damper at *a* = 6.56 m for mode 2.

A selection of the free decay tests is shown in Figures 8(a), 8(b), and 9(a) for CEC, modes 1, 2, and 3, and *a* = 6.56 m; one test in passive-off mode is depicted in Figure 9(b). The point-to-point logarithmic decrement is evaluated within the free decay phase with *i*_mr_^act^ > 0 A which indicates that *F*_mr_^*des*⁡^ is not constrained by the residual force whereby force tracking is possible and the resulting cable damping is determined by the CEC approach. It is observed that, as for the tests at Empa, *δ* ^mea^ does hardly vary when the desired friction force amplitude *F*_mr_^*des*⁡^ (9) can be tracked by the actual friction force amplitude *F*_mr_^act^. This is achieved for modes 1, 2, and 3 as seen from Table 1 which proves that the CEC approach does not only lead to amplitude but also lead to almost frequency independent cable damping as intended (see Section 2). The comparison of ${\overset{-}{\delta }}^{\text{mea}}$ to 0.8*δ* ^theo^, which is the expected value for a cable with flexural rigidity and fixed supported ends [39], yields *η* > 100% which is physically impossible. In contrast, the numbers of *η* which are derived with *δ* ^theo^ = *πa*/*L*, that is, for a cable without flexural rigidity and simply supported ends, are more reasonable. It is therefore concluded that the provisional anchors of this test cable did allow the rotation whereby the cable anchors behaved more like simply supported ends than fixed supported ends.

According to [40], the mode shapes at approximately 6.65 m/228 m ≈ 2.88% of *L* of a cable with flexural rigidity and fixed supported ends and a cable without flexural rigidity and simply supported ends differ by approximately 23.8%. Subtracting 23.8% from the values of *η* listed in Table 1 leads to values in the range between 60% and 75% which is close to the efficiency obtained at the Empa test cable (Section 3.3). Thus, the force tracking errors in the Sutong MR damper are of the same order as those in the laboratory scale rotational MR damper on the Empa cable.

The logarithmic decrement is also determined within the section *i*_mr_^act^ = 0 to estimate the cable damping when *F*_mr_^*des*⁡^ cannot be tracked by the MR damper due to the residual force constraint. This evaluation is constrained to ${\ddot{X}}_{c}\geq 1.5\;\text{m}/{\text{s}}^{2}$ to avoid scattering effects in *δ* ^mea^ due to measurement noise in ${\ddot{x}}_{c}$. The values of *δ* ^mea^ within this range are between 4% and 7% which agree well with the values obtained with passive-off MR damper (Figure 9(b)). The suboptimal but not small values of *δ* ^mea^ at 0 A result from the proper design of the passive MR damper viscous coefficient *c*_mr_ of approximately 24.7 kNs/m (see Figure 2) that avoids clamping effects whereby cable vibrations are also mitigated during a breakdown of the mains and accumulators at the same time.

### 4.2. Cable Damping Tests on Sutong Bridge

#### 4.2.1. Layout of Stay Cable Damping System

**Figure 10:**
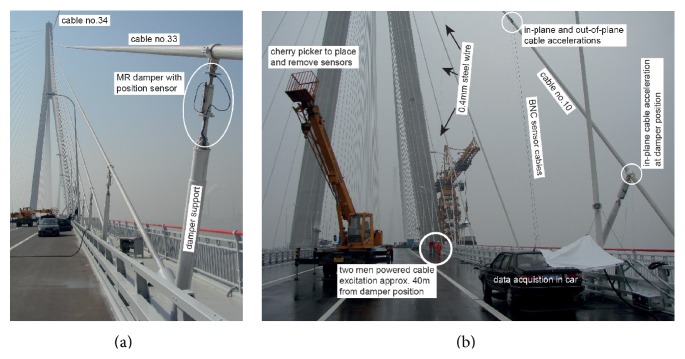
Sutong Bridge: CEC controlled MR damper with position sensor (a); cable excitation by 0.4 mm steel wire approximately 40 m from damper position (b).

The bridge owner decided to mitigate the longest six stay cables, that is, cable number 29 with *L* = 483 m and *f*_1_ ≈ 0.28 Hz to cable number 34 with *L* = 543 m and *f*_1_ ≈ 0.25 Hz, by CEC controlled MR dampers which gives in total 48 CEC controlled MR dampers on the bridge. Always 12 MR dampers are connected to one centralized real-time controller with 12 current drivers. Each MR damper is controlled in a single control loop according to the collocated position sensor that is mounted between MR damper cylinder and cable whereby *x*_*a*_ is measured (Figure 10(a)). The MR damper force range was model-based designed to be able to mitigate cable amplitudes of mode 1 of up to *L*/1700 according to the fib bulletin 30 [9]. Cables number 10 to number 28 are equipped with passive viscous dampers whose viscous damper coefficient was designed to modes 1, 2, and 3 adopting the design presented in [14] that minimizes the damper position (Figure 10(b)). According to the owner specifications, the cables are mitigated by one damper in in-plane direction at approximately 2.3% of the cable length whereby the damper supports did not become too tall (Figure 10(a)).

#### 4.2.2. Measured Dry Wind Induced Stay Cable Vibrations

**Figure 11:**
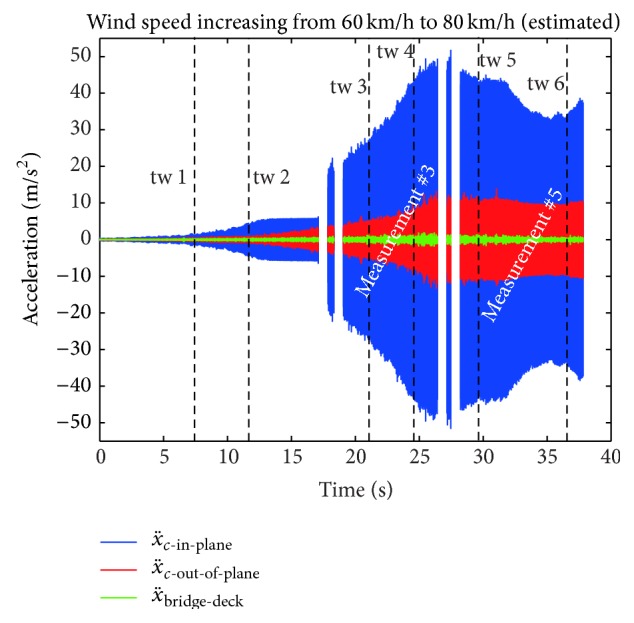
Dry wind induced stay cable vibration on Sutong Bridge, China.

During the installation and testing of the stay cable dampers on the Sutong Bridge dry wind induced stay cable vibrations occurred [44] and were recorded by the authors.  Figure 11 shows the time history of the measured cable accelerations in in-plane and out-of plane directions and the vertical bridge deck acceleration while the wind speed increased very slowly from estimated 60 km/h to estimated 80 km/h [45–47]. The time histories include gaps when the data acquisition had to be stopped and restarted to adjust the maximum voltage threshold of the input channels to ensure maximum resolution of the acquired data.

**Figure 12:**
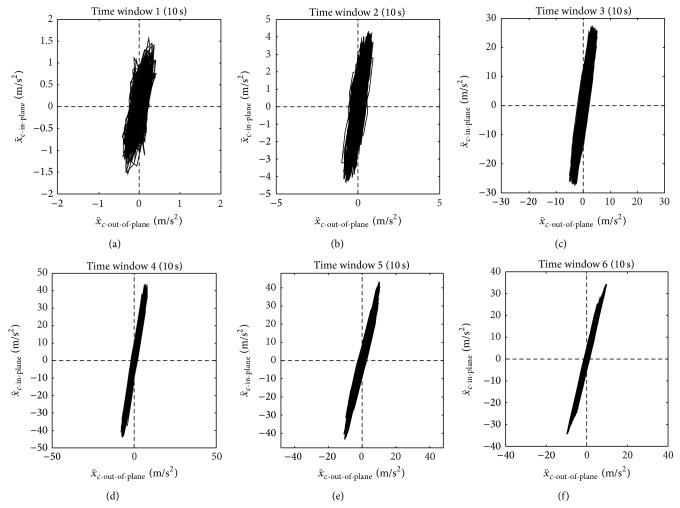
Dry wind induced stay cable vibration on Sutong Bridge, China: in-plane versus out-of-plane cable accelerations during 10 s of time windows 1 to 6 (a–f).

**Figure 13:**
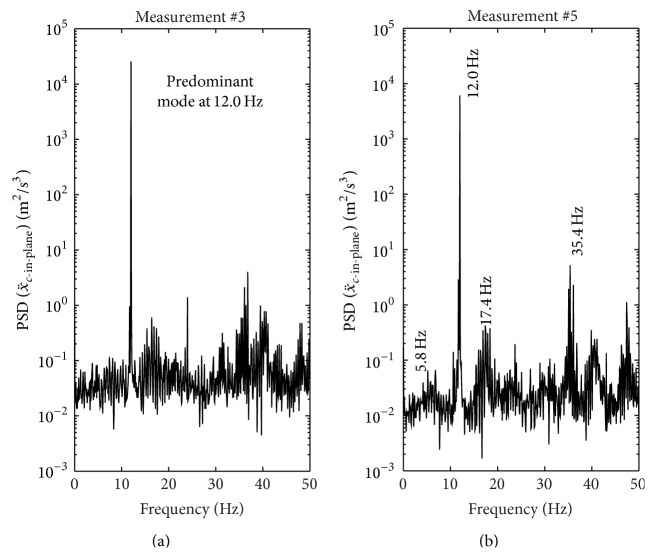
PSDs of in-plane acceleration of measurement #3 (a) and measurement #5 (b).

The ratio between in-plane and out-of-plane amplitudes is quantified during the time windows (tw) 1 to 6 of 10 s each and visualized in Figure 12 by plotting the in-plane acceleration ${\ddot{x}}_{c\text{-in-plane}}$ versus the out-of-plane acceleration ${\ddot{x}}_{c\text{-out-of-plane}}$ with the same scaling on and same dimensions of the *x*- and *y*-axes as done in [48]. In contrast to [48] where modes 2 and 3 were vibrating at the same time and the acceleration data is double-time integrated to estimate cable amplitudes whereby the noise in the in- and out-of-plane signals is suppressed, the acceleration data here is bandpass filtered by a Butterworth filter of order 6 with lower cut-off frequency at 6 Hz and upper cut-off frequency at 17 Hz in order to suppress measurement noise but not attenuate the predominant monoharmonic vibration signal at approximately 12 Hz. As seen from time windows 3 to 6 in Figure 12 the cables on the Sutong Bridge vibrated in one plane, which was not completely the in-plane direction, but the cables did not vibrate spatially which ends up in elliptic shaped trajectories of in- and out-of-plane vibrations as described in [48]. Within the time windows 1 to 6, the amplitude ratios ${\ddot{X}}_{c\text{-in-plane}}/{\ddot{X}}_{c\text{-out-of-plane}}$ are 4.50 (tw 1), 6.67 (tw 2), 6.62 (tw 3), 6.14 (tw 4), 4.29 (tw 5), and 3.96 (tw 6). Looking at the power spectral density estimates (PSDs) of ${\ddot{x}}_{c\text{-in-plane}}$ derived from the data of measurement #3 (Figure 13(a)) and measurement #5 (Figure 13(b)), it is observed that the cable during measurements #3 predominantly vibrated in one mode at 12.0 Hz. During the time of measurement #5, the cable still vibrated primarily at 12 Hz, but ${\ddot{X}}_{c\text{-in-plane}}$ is slightly smaller than during measurement #3 because also higher and lower harmonics were present during measurement #5. Considering these PSDs and looking at the ratios ${\ddot{X}}_{c\text{-in-plane}}/{\ddot{X}}_{c\text{-out-of-plane}}$ of tw 3 and tw 5, it is concluded that the cables during this dry wind induced vibration event vibrated in one plane which was almost the in-plane direction (${\ddot{X}}_{c\text{-in-plane}}\approx 6.6{\ddot{X}}_{c\text{-out-of-plane}}$) when primarily one mode was excited, that is, when a resonant vibration occurred. When also other harmonics were present (tw 5, tw 6), that is, no pure resonant vibration event occurred, the cables still vibrated in one plane, but the inclination angle relative to the in-plane direction was slightly larger. Hence, large amplitude cable vibrations occurred predominantly in in-plane direction and oscillated primarily at one frequency since then all disturbing energy excited one mode. This understanding of a typical resonant vibration event is strengthened by the observations on the Franjo Tudjman Bridge in Croatia where a strong spring storm in 2005 led to in-plane cable vibrations of up to 1 m midspan amplitudes [2]. Thus, the in-plane one damper system of the Sutong Bridge can be assumed to be able to mitigate the predominant cable oscillations. It is also understood that two damper systems, as, for example, those installed on the Dongting Lake Bridge [1], the Shandong Binzhou Yellow River Highway Bridge [29], or the Russky Bridge [25], are able to mitigate efficiently also the out-of-plane direction and thereby represent a more robust solution to the problem of unpredictable cable vibrations but are also more costly due to the doubled number of dampers and the more complex damper supports [49, 50].

#### 4.2.3. Test Results

The envisaged goal of the free decay tests on the Sutong Bridge was to identify the cable damping of modes 1, 2, and 3 of selected cables with CEC controlled MR damper, with passive-off MR damper, with passive oil damper, and without damper.* A 0.4 mm steel wire was used to excite the cables by two men pulling* on the steel wire at the pace of the targeted eigenfrequency using a metronome (Figure 10(b)). In order to maximize the damper motion amplitude to ensure that the CEC approach with *i*_mr_^act^ > 0 A was triggered, the* steel wire was placed on the cable as far away from the damper position as possible, that is, approximately 40 m* (Figure 10(b)). When the cable amplitude could not be increased anymore because the excitation power was balanced by the dissipated power in the damper and the inherent cable damping, the steel wire was released and* then remained in hanging manner as a slackline on the stay cable* to minimize additional damping by the steel wire to the cable.

**Figure 14:**
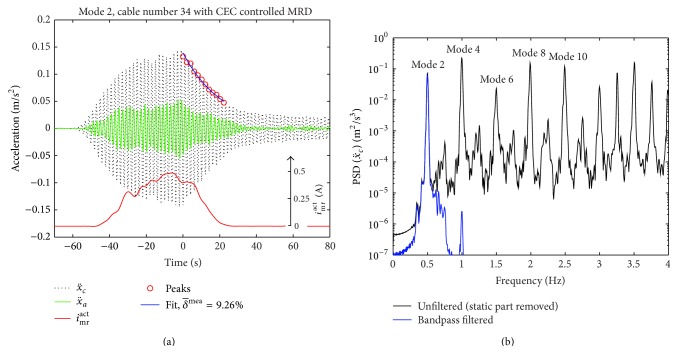
Free decay test of mode 2 with CEC controlled MR damper (a); PSDs of unfiltered and bandpass filtered cable accelerations (b).

**Figure 15:**
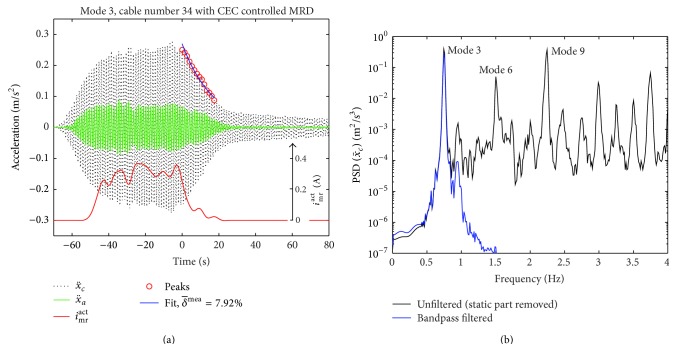
Free decay test of mode 3 with CEC controlled MR damper (a); PSDs of unfiltered and bandpass filtered cable accelerations (b).

The excitation of mode 1 turned out to be impossible because the excitation of mode 1 requires elongating the cord length of the cable for which the two men powered excitation force was too small [51]. Modes 2 and 3 could be excited to sufficient large amplitudes which evoked *i*_mr_^act^ > 0 A in the MR damper due to the CEC approach as seen from Figures 14(a) and 15(a). As seen from Figures 14(b) and 15(b), when the cable was excited by the steel wire at the pace of mode 2 (0.497 Hz), also modes 4, 6, 8, and so forth were excited because the time period of mode 2 is a multiple of the time periods of these modes. Similarly, the excitation of the cable at the pace of mode 3 (0.749 Hz) also triggered modes 6, 9, 12, and so forth since the time period of mode 3 is a multiple of the time periods of these modes. The undesired excitation of these higher harmonics evokes additional local peaks in the free decay responses of the targeted modes, that is, modes 2 and 3. In order to be able to estimate the damping of the targeted modes from the free decay curve by the logarithmic decrement method, the raw data must therefore be filtered for the targeted modal component. The applied filter is a Butterworth filter of order 6 where the lower and upper cut-off frequencies are selected as the frequency of the targeted mode ±0.12 Hz to generate a sharp filter. The PSDs of the bandpass filtered accelerations shown in Figures 14(b) and 15(b) prove that this sharp filter design leads to a stable filter, the filtered signal only includes the modal component of interest, and its magnitude is hardly attenuated. Thus, the time records in Figures 14 and 15 show the true acceleration amplitudes of modes 2 and 3, respectively. The mean logarithmic decrement ${\overset{-}{\delta }}^{\text{mea}}$ of the cable is then determined from the exponential fit of the peaks of the bandpass filtered cable acceleration during the free decay phase.

The damping efficiency *η* (11) is determined assuming a cable with typical flexural rigidity and fixed supported ends, that is, *η* computed with *δ* ^theo^ ≈ 0.8*πa*/*L* [39], and assuming a cable without flexural rigidity and simply supported ends, that is, *δ* ^theo^≅*πa*/*L* used in (11) [12]. As Table 2 shows, both resulting damping efficiencies are physically impossible for controlled damping without negative stiffness; only damping combined with negative stiffness can generate more damping which is limited to approximately twice the value of  (3) [32]. One reason for the too high *η* may be the additional damping that is generated both by the steel wire and the BNC sensor cables that were hanging on the cable as slacklines during the free decay; note that this cannot be avoided. Another explanation may be the energy spillover to higher modes that is visible in the PSDs of Figures 14(b) and 15(b) which may have been caused by not pure single harmonic excitation and/or the nonlinear control force of the CEC approach (9). The energy transfer to higher modes acts as an energy sink for the bandpass filtered signal of ${\ddot{x}}_{c}$ whereby ${\ddot{x}}_{c}$ decays faster and ${\overset{-}{\delta }}^{\text{mea}}$ becomes higher than expected for controlled damping without negative stiffness.

## 5. Conclusions

This paper first describes the cycle energy control (CEC) approach for the mitigation of stay cables with MR dampers. The CEC approach is designed to generate amplitude and almost frequency independent cable damping since both actual amplitude and actual mode of vibration depend on the actual unknown wind conditions whereby these two quantities are unpredictable. Tests on a single strand cable and a stay cable of the Sutong Bridge confirm that amplitude and almost frequency independent cable damping is achieved. The discussion of the results indicates that the experimentally obtained cable damping values are close to the theoretical value due to optimal linear viscous damping of a taut string if the cable anchors behave as simply supported ends and force tracking errors in the actual MR damper force are negligibly small.

The implementation of the CEC approach on the Sutong Bridge, China, is briefly described. A real dry wind excited cable vibration event on the Sutong Bridge is described and analysed. The measurements show that in-plane amplitudes are more than 6 times greater than out-of-plane amplitudes if predominantly one mode is vibrating and 4 times greater if also other harmonics are present. Hence, the one stay cable damper system of the Sutong Bridge can mitigate at least 80% of the cable vibration energy. The evaluation of the free decay tests of CEC controlled stay cables on the Sutong Bridge yields damping values that are higher than the expected from optimal linear viscous damping of a taut string which is physically impossible for controlled damping without negative stiffness. This seemingly impossible result is interpreted to be caused by additional damping introduced by the excitation steel wire and the BNC sensor cables that were hanging as slacklines on the stay cable and by energy spillover to higher modes which acts as energy sink.

The discussion of the experimentally obtained cable damping values reveals that their interpretation requires taking into consideration many factors such as cable support conditions and force tracking errors in the MR damper and additional energy sinks due to energy spillover to higher modes and excitation and sensor cables hanging on the cable whereby the interpretation of the measured cable damping values becomes a complex task.

## Figures and Tables

**Table 1:** Measured mean logarithmic decrement due to CEC.

*a* (m)	Mode	${\overset{-}{\delta }}^{\text{mea}}$ (%)	0.8δ^theo^ (%)	δ^theo^ (%)	η (%)
5.95	1	6.94	6.56	8.20	84.63
	2	7.68	6.56	8.20	93.66
	3	7.97	6.56	8.20	97.20

6.56	1	7.49	7.23	9.04	82.85
	2	7.75	7.23	9.04	85.73
	3	8.11	7.23	9.04	89.71

7.81	1	10.44	8.61	10.76	97.03
	2	10.19	8.61	10.76	94.70
	3	10.63	8.61	10.76	98.79

**Table 2:** Measured mean logarithmic decrement due to CEC on Sutong Bridge.

*a*/*L* (%)	Mode	${\overset{-}{\delta }}^{\text{mea}}$ (%)	$\eta ={\overset{-}{\delta }}^{\text{mea}}/\left(0.8\pi \left(a/L\right)\right)$ (%)	$\eta ={\overset{-}{\delta }}^{\text{mea}}/\left(\pi (a/L)\right)$ (%)
2.3	2	9.26	160.2	128.2
	3	7.92	137.0	109.6
